# Collaterals: An Important Determinant of Prolonged Ischemic Penumbra Versus Rapid Cerebral Infarction?

**DOI:** 10.3389/fneur.2014.00208

**Published:** 2014-10-14

**Authors:** Elisabeth Breese Marsh, Richard Leigh, Martin Radvany, Philippe Gailloud, Rafael H. Llinas

**Affiliations:** ^1^Department of Neurology, The Johns Hopkins University School of Medicine, Baltimore, MD, USA; ^2^Department of Neurology, Johns Hopkins Bayview Medical Center, Baltimore, MD, USA; ^3^Department of Radiology, The Johns Hopkins University School of Medicine, Baltimore, MD, USA

**Keywords:** penumbra, ischemic stroke, collaterals, intra-arterial thrombolysis, recovery

## Abstract

Intravenous tissue plasminogen activator is the mainstay for the treatment of acute ischemic stroke in patients presenting within 4.5 h of symptom onset. Studies have demonstrated that treating patients early leads to improved long-term outcomes. MR imaging currently allows quantification of the ischemic penumbra in order to better identify individuals most likely to benefit from intervention, irrespective of “time last seen normal.” Its increasing use in clinical practice has demonstrated individual differences in rate of infarction. One explanation for this variability is a difference in collateral blood flow. We report two cases that highlight the individual variability of infarction rate, and discuss potential underlying mechanisms that may influence treatment decisions and outcomes.

## Introduction

Intravenous tissue plasminogen activator (IV tPA) is approved by the FDA for the treatment of acute ischemic stroke for patients presenting within 3 h of symptom onset. Additional benefit has been shown within the 4.5 h window (1, 2). Subsequent studies have demonstrated improved outcomes for patients treated early, indicating that time is an important factor in determining the success of thrombolysis (3). This is most likely because vessel occlusion results in a poorly perfused ischemic core those infarcts rapidly, with a larger area of marginally perfused tissue, or penumbra (4). Neurons within the hypoperfused area are unable to maintain a resting potential, resulting in clinical dysfunction, but are not yet irreversibly damaged (5). The time that is required for the ischemic core to expand to match the penumbra is variable from patient to patient. One study showed that 91% of patients had a favorable diffusion (core) to perfusion (penumbra) mismatch on neuroimaging within 3 h from “last seen normal” falling to 72% by 3–6 h post-symptom onset (6, 7). Importantly, a small group of patients continued to display a favorable mismatch up to 24 h from symptom onset. We present two cases that demonstrate this individual variability of infarct progression.

## Case Presentation

### Case 1

**Figure 1:**
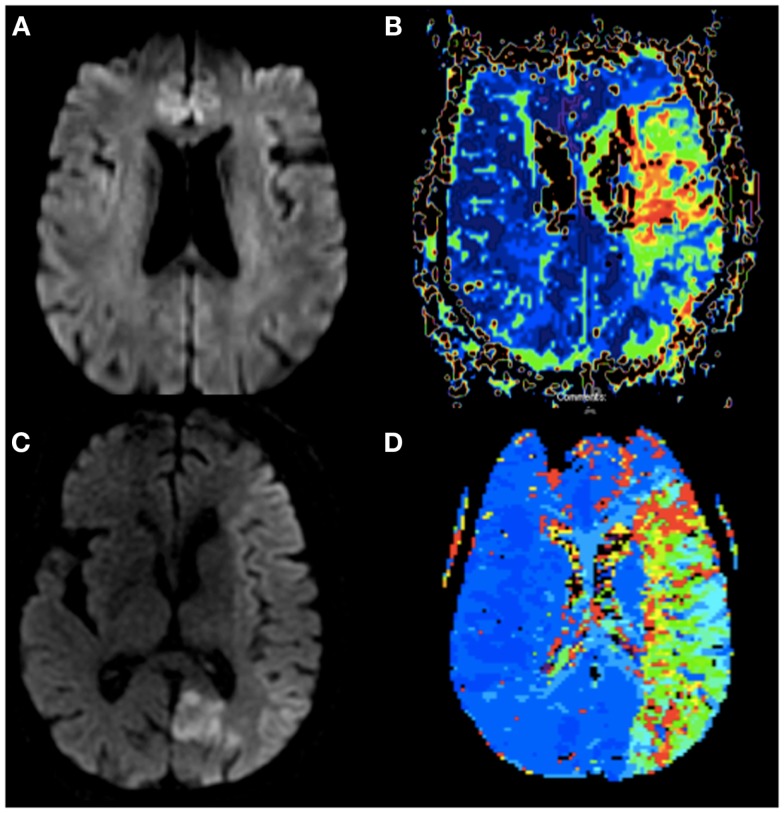
**(A)** Diffusion weighted imaging of Case 1 without evidence of infarction 16 h after onset of symptoms. **(B)** Perfusion weighted imaging (TTP) of Case 1 showing patchy hypoperfusion of the left MCA. **(C)** Diffusion weighted imaging of Case 2 <60 min from stroke onset with early changes throughout the entire left MCA territory and her prior subacute left PCA infarct. **(D)** Perfusion weighted imaging (TTP) of Case 2 showing hypoperfusion of the entire left MCA.

**Figure 2:**
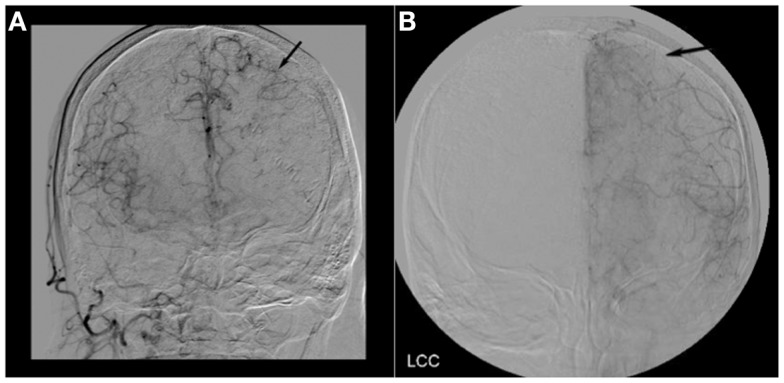
**(A)** Cerebral angiogram of Case 1 showing robust collateral flow through the pial vessels in the late arterial phase (black arrow). **(B)** Cerebral angiogram of Case 2 showing a lack of collateral flow through the pial vessels in the late arterial phase (black arrow).

WS is a 70-year-old man with a history of hypertension, hyperlipidemia, and diabetes, who presented with aphasia and a right hemiparesis. He was last seen normal by his wife at 9 p.m. the evening prior to admission. When she came to bed at 2 a.m. she noted that he could not move his right side or communicate, and called EMS. He arrived in the Emergency Department at 2:34 a.m. His blood pressure was 121/60 mmHg. Serum glucose was 126. He was noted to be in atrial fibrillation. His troponin was elevated at 7.12 with mild ST segment changes. His NIH stroke scale was 12, with points given for disorientation, gaze preference, dysarthria, aphasia, and hemiparesis. He was outside of the window for treatment with IV tPA. Work-up was initiated. He vomited during CT angiogram, requiring intubation for airway protection. Coffee ground emesis was also noted. Cerebral perfusion was maximized with fluids and positioning and he was admitted to the Neurocritical Care Unit for further monitoring. Permissive hypertension was allowed as per our institution’s policy regarding treatment of acute stroke; however, his systolic blood pressures remained around 120 mmHg. He did not exhibit evidence of perfusion dependence on examination so hypertensive therapy was not pursued. Due to medical instability, an MRI of the brain was not performed until the following morning. Neuroimaging revealed a large perfusion deficit encompassing much of the left middle cerebral artery (MCA) territory, matching his clinical deficits, with no clear diffusion abnormality (Figures 1A,B). He remained symptomatic, and the decision was made to proceed with intra-arterial intervention given the lack of infarcted tissue. Over 16 h after being last seen normal, successful recanalization was achieved using the penumbra clot retrieval device. Angiography demonstrated robust collateral flow through the pial vessels (Figure 2A). Follow-up MR imaging showed only a small area of diffusion restriction. His aphasia and hemiparesis markedly improved after recanalization, and 4 days later he walked out of the hospital with no rehabilitation needs.

### Case 2

LD is an 86-year-old woman with a history of hypertension, hyperlipidemia, and recurrent breast cancer, who presented with right sided weakness and vision loss. She was last seen normal the night prior to admission and was therefore not an IV tPA candidate. An MRI of the brain showed diffusion restriction within the left posterior cerebral artery territory. A CT angiogram showed mild atherosclerotic changes. She was awaiting a transthoracic echocardiogram prior to discharge and recovering well until hospital day 3. At 9:40 a.m. she walked to the bathroom unassisted, but was found 3 min later by the neurology team to be aphasic, with left gaze deviation, and a dense right hemiparesis. Her NIH stroke scale was 19. A head CT showed no intracranial hemorrhage, but a hyperdense left MCA sign. Acute ischemia was suspected. Given her recent PCA stroke she was again not a candidate for IV tPA. A hyperacute MRI was completed <60 min after the onset of symptoms to determine if she was a candidate for intra-arterial intervention. Despite the short time between symptom onset and imaging, a significant area of new restricted diffusion involving mainly the cortex was noted within the left hemisphere, along with a larger perfusion deficit and cut off of the M1 branch of the left MCA (Figures 1C,D). Given the acute onset of symptoms and presence of a diffusion/perfusion mismatch, the decision was made to proceed with intervention. Angiography demonstrated a lack of collaterals without significant flow through the pial vessels (Figure 2B). Unfortunately, despite complete recanalization within 4 h of symptom onset, a large portion of the MCA territory was infarcted on follow-up imaging. She remained aphasic and densely hemiparetic through to discharge to a rehabilitation facility.

## Discussion

MRI and CT perfusion allow real-time quantification of infarcted versus hypoperfused tissue; however, the trials looking at the use of MR imaging to predict who will benefit from late recanalization have been mixed (8–12). Therefore, time from symptom onset continues to be the standard indicator of which patients will benefit most from recanalization. Here, we describe two cases that illustrate the individual variability in the time course of infarction between patients presenting with large vessel occlusion. In Case 1, time of recanalization was prolonged from stroke onset, yet irreversible damage was minimal. Additional cases have been reported in the literature, with viable tissue being documented up to 17 h from stroke onset (13–15). In Case 2, after only 60 min, a significant portion of the vascular territory had already undergone infarction. In both cases, response to treatment paralleled imaging characteristics, but did not parallel the outcomes predicted by time alone.

The increased availability of MRI and advent of perfusion imaging techniques provide an opportunity to identify patients with a “favorable ischemic profile” independent of time from stroke onset (12). This favorable profile appears more common in the hyperacute setting, but as illustrated above, prolonged mismatches may occur. Conversely, unfavorable mismatches can also occur after only a short period of vessel occlusion (16). Correctly identifying both groups may help to tailor therapy beyond current guidelines. Studies looking for the favorable profile have, to date, yielded mixed results (8–12). It is likely that we simply have yet to consistently identify the right subgroup who will benefit, and/or have failed to account for an additional critical unmeasured variable. One reason that studies like MR RESCUE (8) have been disappointing may be because perfusion weighted imaging likely does not adequately take into account collateral blood flow, and that slowed flow is not the same as absent flow. In MR RESCUE, the information obtained was “presence of a mismatch,” but at no point was comment made regarding presence of favorable collateral blood flow on angiogram, which we believe would have been useful information in deciding whether to move forward with recanalization. Though these studies have failed to give rise to definitive criteria that reliably predict good long-term outcome, they have confirmed the presence of a select group who continue to have significant diffusion/perfusion mismatches far outside the accepted 4.5 h window (12). While it is statistically more likely for a favorable mismatch to occur early, failing to account for these individuals would result in a missed opportunity for intervention given their prolonged therapeutic window (6, 17).

In an elegant set of experiments, Jones et al. showed that with a reduction of cerebral blood flow (CBF) in an animal occlusion model, the amount of reduced flow predicted irreversible infarction. They found that reduction of local CBF in the range of 23 cc/100 gm/min resulted in reversible paralysis without infarction once blood flow was reinstated. Of interest, reduction of local CBF further to 10–12 cc/100 gm/min for 2–3 h resulted in irreversible infarction (18). In humans with acute stroke, the specific factors that determine rate of infarction and the local CBF remain unclear; however, the primary contributor is likely the presence versus absence of viable collaterals. Collateral flow through the pial vessels was clearly evident on the angiogram in our “late infarcter” (Case 1), but poorly visualized in our “early infarcter” (Case 2) (19). Prior studies looking at both digital subtraction angiography (DSA) and CT angiography in predicting outcomes of thrombolysis have shown similar results, confirming that those with better long-term outcomes typically exhibit more robust flow through the collaterals (20, 21).

If rate of infarction is dependent on the presence of viable collaterals, it is important to consider factors that may influence collateral flow. Collaterals may be seen in response to acute vascular occlusion, or as the result of chronic stenosis of a single vessel (i.e., Moyamoya syndrome). Patients who are younger and those with a lower atherosclerotic burden may have the ability to muster better acute collateral flow (22). When relatively healthy pial vessels are successfully recruited following an MCA occlusion, the resulting infarct often spares much of the cortex, rather than involving the entire vascular territory (23, 24). Similarly, vascular stenosis of a single large artery stimulates chronic collateralization within the vascular bed, making the specific territory more resistant to ischemia should the vessel occlude.

In addition to collateral blood flow, when there is a chronic low-flow state or vascular stenosis, ischemic preconditioning may occur. This phenomenon was first described in the cardiac literature and has been well demonstrated in MCA occlusion models (25). In humans, it has been observed that patients undergoing balloon occlusion, and those with prior transient ischemic attacks, have smaller strokes when the same vascular distribution is subsequently compromised (26, 27). It seems that brain exposed to chronic ischemia (i.e., large vessel stenosis) is better able to tolerate future ischemic events due to physiologic and molecular changes within the affected neurons (25).

Our cases, in the context of these prior studies, support the concept that the combination of robust collateral blood flow (either acutely or chronically) and some degree of existing ischemic preconditioning, in conjunction with other factors such as the metabolic demand of the at risk tissue (with gray matter areas being most at risk) and systolic blood pressure affecting overall cerebral perfusion, results in patients who are “late infarcters” (28). In Case 1, following clot retrieval it was apparent that WS had some degree of pre-existing MCA stenosis. This likely led to chronic collateralization, as evidenced by robust flow through his pial vessels. Unfortunately, the presence of favorable collaterals and prior ischemic preconditioning are not variables that can be easily determined on current MR imaging. Accordingly, they may be the critical variables missing from the prior “late thrombolysis” studies.

For the population as a whole, time remains the single most important variable used to predict who will benefit from thrombolysis. However, with advances in neuroimaging, a surrogate marker that allows better individualization of treatment would be ideal. Though expansion of the ischemic core likely occurs over a fairly predictable time course, we have shown two cases that illustrate significant variability. A better understanding of the role of collateral blood flow and ischemic preconditioning may allow us to better identify those individuals who may be “late infarcters,” or even develop strategies to slow infarction rate.

## Conflict of Interest Statement

The authors declare that the research was conducted in the absence of any commercial or financial relationships that could be construed as a potential conflict of interest.
